# Alternate expression of *CONSTANS-LIKE 4* in short days and *CONSTANS* in long days facilitates day-neutral response in *Rosa chinensis*

**DOI:** 10.1093/jxb/eraa161

**Published:** 2020-03-28

**Authors:** Jun Lu, Jingjing Sun, Anqi Jiang, Mengjuan Bai, Chunguo Fan, Jinyi Liu, Guogui Ning, Changquan Wang

**Affiliations:** 1 College of Horticulture, Nanjing Agricultural University, Nanjing, China; 2 College of Horticulture and Forestry Sciences, Huazhong Agricultural University, Wuhan, China; 3 Shanghai Jiao Tong University, China

**Keywords:** Continuous flowering, day-neutral plants, long-day plants, photoperiod responses, *Rosa chinensis*, short-day plants

## Abstract

Photoperiodic flowering responses are classified into three major types: long day (LD), short day (SD), and day neutral (DN). The inverse responses to daylength of LD and SD plants have been partly characterized in Arabidopsis and rice; however, the molecular mechanism underlying the DN response is largely unknown. Modern roses are economically important ornamental plants with continuous flowering (CF) features, and are generally regarded as DN plants. Here, *RcCO* and *RcCOL4* were identified as floral activators up-regulated under LD and SD conditions, respectively, in the CF cultivar *Rosa chinensis* ‘Old-Blush’. Diminishing the expression of *RcCO* or/and *RcCOL4* by virus-induced gene silencing (VIGS) delayed flowering time under both SDs and LDs. Interestingly, in contrast to *RcCO*-silenced plants, the flowering time of *RcCOL4*-silenced plants was more delayed under SD than under LD conditions, indicating perturbed plant responses to day neutrality. Further analyses revealed that physical interaction between RcCOL4 and RcCO facilitated binding of RcCO to the CORE motif in the promoter of *RcFT* and induction of *RcFT*. Taken together, the complementary expression of *RcCO* in LDs and of *RcCOL4* in SDs guaranteed flowering under favorable growth conditions regardless of the photoperiod. This finding established the molecular foundation of CF in roses and further shed light on the underlying mechanisms of DN responses.

## Teaser

The complementary expression of *RcCO* in long days and *RcCOL4* in short days guarantees flowering under favorable growth conditions regardless of the photoperiod, thus facilitating the day-neutral response of roses.

## Introduction

Flowering is a biological process indicating the shift from vegetative growth to reproductive development; as such, its accurate timing is key to reproduction and survival. The timely transition from vegetative to floral meristems in higher plants is programmed by external environmental cues and endogenous signals (Langridge, 1957). So far, six genetic pathways have been identified to control plant flowering, namely photoperiod, vernalization, ambient temperature, gibberellin, age, and autonomous pathways. All these pathways finally converge on the common downstream flowering integrators *FT* (*FLOWERING LOCUS T*) and *SOC1* (*SUPPRESSOR OF OVEREXPRESSION OF CO1*), whose expression leads to the induction of floral identity genes such as *LFY* (*LEAFY*) and *AP1* (*APETALA1*), followed by flower bud formation and burst (Fornara *et al.*, 2010; Srikanth and Schmid, 2011).

The photoperiod pathway refers to the regulation of flowering in response to daylength. Based on their daylength requirements, plants are classified as long day (LD), short day (SD), or day-neutral (DN) (Jeong and Clark, 2005; Srikanth and Schmid, 2011). Arabidopsis is one of the well-known LD plants and flowers much earlier under LD than SD conditions. In contrast, rice is considered a SD plant that flowers faster in SDs than in LDs. The DN plants flower interdependently of the photoperiodic conditions. The inverse responses to daylength observed between Arabidopsis (LD plant) and rice (SD plant) are partly explained by the difference between the function of CO in Arabidopsis and the rice homolog *HEADING DATE 1* (*Hd1*) (Izawa *et al.*, 2002; Ryosuke *et al.*, 2003). A common role of GIGANTEA (GI)–CONSTANS (CO)–FLOWERING LOCUS T (FT) in Arabidopsis and rice has been demonstrated (Izawa *et al.*, 2002; Ryosuke *et al.*, 2003). *Hd1* promotes the flowering under SD conditions and inhibits it under LD conditions in rice, whereas *CO* only accelerates flowering under LDs in Arabidopsis. Further study proves *Hd1* can up-regulate *Hd3a* (the homolog of Arabidopsis *FT* in rice) expression preferably under SD conditions, and the Hd1–Hd3a pathway in rice mimics the CO–FT model in Arabidopsis (Yano *et al.*, 2000; Reina *et al.*, 2008; Xue *et al.*, 2009). However, the DN response is the most poorly characterized among the three types of photoperiodic flowering responses. In the DN plant tomato (*Solanum lycopersicum*), the universal florigenic signal triggered by *SFT* (*SINGLE FLOWER TRUSS*), a *FT* homolog, is a known flowering inducer under different daylengths (Lifschitz and Eshed, 2006). Consistently, the florigen gene *ZEA CENTRORADIALIS8* (*ZCN8*) in maize (*Zea mays*) is associated with the floral transition both in DN temperate maize and in SD-requiring tropical maize, and has been shown to be regulated by different chromatin modifications at the floral transition (Lazakis *et al.*, 2011).

Roses are economically important ornamental plants with high symbolic value and great cultural importance all over the world. They are extensively used as garden plants, cut flowers, as well as potted flowers, and also for the production of essential oils in the cosmetic industry (Bendahmane *et al.*, 2013). Overall, there are three different flowering modes in rose plants, once-flowering (OF) genotypes (such as *Rosa multiflora*), continuous flowering (CF) genotypes (such as *Rosa chinensis* cv ‘Old Blush’) which flower during the growing seasons, and occasionally re-blooming (OB) genotypes, such as the vegetative mutants of the CF genotype ‘Pompon de Paris Climbing’ (Bendahmane *et al.*, 2013; Kurokura *et al.*, 2013). In OF genotypes, floral transition occurs during short photoperiods in early spring. In CF genotypes, floral transition occurs during short photoperiods and long photoperiods, such as in late spring and summer, and as such they are generally considered as DN plants or photoperiod-insensitive plants. The comparison of OF and CF varieties presents a unique opportunity to investigate the DN photoperiod pathway in roses.

*Rosa chinensis* cv ‘Old Blush’ is one of the important progenitors of modern rose cultivars and is regarded as the main contributor of recurrent flowering (Martin *et al.*, 2001; Bendahmane *et al.*, 2013). In a recent study of rose plants, *KSN*, a *TFL1* homolog of Arabidopsis, is shown to act as a floral repressor. In OF rose cultivars, *KSN* is repressed in winter and early spring under short photoperiods, so they bloom only once in spring. After blooming, *KSN* expression is activated by an as yet unknown mechanism which in turn represses further flower formation in long-photoperiod summer (Iwata *et al.*, 2012; Randoux *et al.*, 2012; Bendahmane *et al.*, 2013). It is further established that the 10 kb insertion of a copia retrotransposon in the second intron of the *KSN* gene in the CF rose ‘Old Blush’ blocks the expression of *KSN* and enables its flowering regardless of daylength (Iwata *et al.*, 2012). Interestingly, studies in a different genetic background using distinct mapping populations have identified two quantitative trait loci (QTLs) for continuous flowering, suggesting that the CF trait may be under the control of multiple regulators (Dugo *et al.*, 2005; Shubin *et al.*, 2015). In the present study, *RcCO* and *RcCOL4* were identified as floral activators up-regulated under LD or SD conditions, respectively, in ‘Old Blush’. RcCOL4 physically interacted with RcCO and thereby facilitated RcCO binding to the promoter of *RcFT* to activate its transcription. The complementary expression of these two positive floral regulators in LDs and SDs guaranteed rose flowering under favorable growth conditions irrespective of the photoperiod. This finding provided a molecular model of the CF trait in rose and deciphered the underlying mechanism of the DN response.

## Materials and methods

### Plant materials and growth conditions

OF roses *Rosa laevigata*, *Rosa berberifolia*, and *Rosa multiflora*, and CF roses *Rosa chinensis* cv ‘Old Blush’, *R. chinensis* cv ‘Sichun’, *R. chinensis* cv ‘Viridiflora’, and *Rosa hybrida* cv ‘Molde’ were grown in the rose resource nursery of Nanjing Agricultural University. Cuttings or explants were collected from multistemmed stock plants, and the generated cutting plants or seedling *in vitro* were used for the present experiment. Plants propagated from cuttings were used for flower phenotyping, and were grown in plant incubators with controlled conditions (25 °C, 40% relative humidity, and 200 μmol m^–2^ s^–1^) under SDs (8:16 h, light:dark) or LDs (16:8 h, light:dark). Seedlings *in vitro* of *R. chinensis* cv ‘Old blush’ were used as starting materials for *in vitro* propagation, and were repeatedly subcultured every 3–4 weeks on proliferation medium [Murashige and Skoog (MS)+1.5 mg l^–1^ 6-benzyladenine (6-BA)+0.1 mg l^–1^ 1-naphthaleneacetic acid (NAA)+30 g l^–1^ sucrose+6.5 g l^–1^ agar, pH 5.75]. The resultant young seedlings were then used as transient transformation materials.

Arabidopsis plants were also grown in a plant incubator with controlled conditions (22 °C, 40% relative humidity, and 180 μmol m^–2^ s^–1^) under LDs (16:8 h, light:dark).

### For the phylogenetic analysis

First, the hidden Markov model of the BBX domain (PF00643) t from the Pfam database (http://pfam.xfam.org/) was used to retrieve all of the candidate members of the BBX gene family from *R. chinensis*, *R. multiflora*, and Arabidopsis by using the HMMER v3.0 program with default parameters. Then, all candidate protein sequences were further validated on InterPro (http://www.ebi.ac.uk/interpro/) and SMART (http://smart.embl-heidelberg.de/) for the integrity of their conserved domains. Multiple sequence alignments were executed by using MAFFT v7.409 (Katoh and Standley 2013) with the L-INS-I alignment strategy (most accurate). Systematic phylogenetic analysis and maximum-likelihood phylogenetic trees were constructed using FastTree software with the JTT+CAT model (http://www.microbesonline.org/fasttree/) (Price *et al.*, 2009). The phylogenetic trees were visualized and edited using MEGA7 software (https://www.megasoftware.net/home) (Kumar *et al.*, 2016). To explore the domain compositions of the full-length sequences of BBX domain-containing proteins, SMART (https://smart.embl-heidelberg.de/) and Pfam (https://pfam.xfam.org/) online programs were used to identify all the conserved domains with default parameters.

### Plasmid constructions

The overexpression constructs were prepared by amplifying *RcCO* (*RcChr2g0164091*) and *RcCOL4* (*RcChr6g0299051*) from the cDNA of ‘Old Blush’ using the primers listed in Supplementary Table S1 at *JXB* online. Subsequently, PCR products were cloned into the pENTR-D-TOPO entry vector (Invitrogen) and then cloned into the binary vector pFAST-R05 (http://www.psb.ugent.be/).

For virus-induced gene silencing (VIGS), gene-specific fragments of *RcCO* and *RcCOL4* were amplified using the primers listed in Supplementary Table S1 and then cloned into pTRV2 to generate the VIGS constructs.

For protein–protein interaction analysis by rose transient assay, coding sequences of *RcCO* and *RcCOL4* were inserted into pMK7-nL-WG2 or pMK7-cL-WG2 (http://www.psb.ugent.be/), respectively, by LR reaction. For promoter binding analysis, a fragment containing 1976 bp upstream of the translational start site of *RcFT* (*RcChr4g0439111*) was amplified from the genome sequence. Next, the PCR product was cloned into the pENTR-D-TOPO vector and subsequently recombined into pGBWL7 (http://www.psb.ugent.be/).

For yeast two-hybrid experiments, full coding sequences of *RcCO* and *RcCOL4* were inserted into the pGBKT7 vector (bait, BD) or the pGADT7 vector (prey, AD). For yeast one-hybrid assay used to identify the promoter binding, 1976 bp upstream of the translational start site of *RcFT* was cloned into the pHIS2 vector.

To substitute Cys by Ser in Box1 and Box2 in the *RcCOL4* sequence, we used the Hieff Mut™ Site-Directed Mutagenesis Kit (YEASEN, Shanghai, China) with the base substitution primers listed in Supplementary Table S1.

To induce protein in *Escherichia coli*, the coding sequences of *RcCO* and *RcCOL4* were cloned into pGEX4T-1 using the Hieff Clone^®^ Plus One Step Cloning Kit (YEASEN).

### Gene expression analysis

For RNA isolation, the uppermost young leaves from 40-day-old rose plants propagated from cuttings were harvested and frozen in liquid nitrogen. Total RNA was then extracted using the FastPure Plant Total RNA Isolation Kit (VAZYME, Nanjing, China), and 1 µg of total RNA was reverse transcribed using TransScript One-Step gDNA Removal and cDNA Synthesis SuperMix (TRANSGEN BIOTECH, Beijing, China) according to the manufacturer’s instructions. Quantitative real-time PCR (RT-qPCR) was performed to identify gene expression levels by using TB Green™ Premix Ex Taq™ II (TaKaRa, Dalian, China) and *RcGAPDH* was used as reference gene as described previously (Liu *et al.*, 2018). Every experiment was conducted with three replicates each with three technical repeats. Semi-quantitative RT-PCR was performed to identify gene expression in Arabidopsis by using the primers listed in Supplementary Table S1, and *Actin2* was used as reference gene (Chang *et al.*, 2016).

### Virus-induced gene silencing

For the generation of gene-silenced plants, VIGS was performed as previously reported (Tian *et al.*, 2014). Briefly, the *Agrobacterium tumefaciens* strain GV3101 carrying TRV-*RcCO* or TRV-*RcCOL4* was grown at 28 °C in Yeast Extract Broth medium supplemented with 20 mM acetosyringone, 50 mg l^–1^ gentamicin, 50 mg l^–1^ kanamycin, and 30 mg l^–1^ rifampicin, and shaken on a rocking platform at 250 rpm for ~18–24 h. Subsequently, *Agrobacterium* cells were harvested and suspended in infiltration buffer [10 mmol l^–1^ MgCl_2_, 200 mmol l^–1^ acetosyringone, 10 mmol l^–1^ MES, 0.01% (v/v) Silwet-L77, pH 5.6]. A mixture of *A. tumefaciens* cultures containing pTRV1 and constructed pTRV2-*RcCO*/*RcCOL4* in a ratio of 1:1 (v/v) was adjusted to OD_600_=0.6, and the mixture of pTRV1 and pTRV2 with the same concentration was also prepared as a negative control. Then, *R. chinensis* cv ‘Old Blush’ cuttings were submerged in infiltration buffer and exposed to a vacuum of −25 kPa twice, each for 60 s. The infiltrated cuttings were briefly washed with distilled water and planted in substrates [roseite:perlite:peat soil 1:1:1 (v/v/v)] for further analysis.

### Transient transformation analysis in rose

To perform the protein–protein interaction or promoter binding analysis, transient transformations using young shoots of *R. chinensis* cv ‘Old Blush’ were conducted as previously described (Lu *et al.*, 2017). Briefly, for protein–protein interaction, the *A. tumefaciens* strain GV3101 carrying pMK7-nL-WG2-*RcCO* or pMK7-cL-WG2-*RcCOL4* was co-infiltrated into rose shoots to test the possibility of luciferase reconstitution. For promoter binding analysis, the *A. tumefaciens* strain GV3101 carrying pFAST-R05-*RcCO*/*RcCOL4* and pGBWL7-*pFT* or other different combinations were co-infiltrated into rose shoots to determine changes in activity of luciferase.

### Luciferase imaging

Luciferase imaging was performed using a CCD camera (Andor Technology). At 48 h after the agro-infiltration of young shoots of *R. chinensis* cv ‘Old Blush’ the images were acquired every 10 min for 60 min, and luciferase activity was quantified as mean counts per pixel per exposure time using Andor Solis image-analysis software (Andor Technology).

### Yeast hybrid experiments

For yeast one-hybrid assay, yeast Y187 cells carrying *pFT-pHis* were grown on SD medium lacking tryptophan and histidine (SD/-Trp/-His) with different contents of 3-amino-1,2,4-triazole (3-AT) to optimize the concentration for inhibiting self-activation. After that, pGADT7-*RcCO*/pGADT7-*RcCOL4* and *pFT-pHis* were co-transformed into yeast Y187 cells, and the binding activity was examined on SD medium lacking tryptophan, leucine, and histidine (SD/−Trp/−Leu/−His) with the proper concentration of 3-AT.

For yeast two-hybrid (Y2H) assay, the Y2H yeast strain was used according to the manufacturer’s instructions (Clontech, Mountain View, CA, USA). Yeast transformation was carried out using the lithium acetate method. The Y2H yeast cells containing prey (RcCO-AD) and bait (RcCOL4-BD/RcCOL4M1-BD/RcCOL4M2-BD) were co-cultured on SD medium lacking leucine and tryptophan (SD/−Leu/−Trp). Putative transformants were then transferred to SD medium lacking adenine, histidine, leucine, and tryptophan (SD/−Ade/−His/−Leu/−Trp; Clontech) with or without X-α-gal. At least three independent transformations were performed and three clones per transformations were used to evaluate the protein interaction.

### Electrophoretic mobility shift assay

The EMSA was performed using biotinylated probes (Sangon Biotech, Shanghai, China) and the Light Shift Chemiluminescence EMSA kit (Thermo Scientific, https://www.thermofisher.com/). Briefly, *E. coli* strain BL21 carrying pGEX4T-1-*RcCO* or pGEX4T-1-*RcCOL4* was grown at 37 °C in Luria–Bertani medium supplemented with 100 mg l^–1^ ampicillin and shaken on a rocking platform to OD_600_=0.5. Subsequently, the *E. coli* cultures were supplemented with 500 μmol l^–1^ isopropyl-β-d-thiogalactopyranoside (IPTG) and shaken on a rocking platform at 25 °C to induce the protein expression. The obtained *E. coli* cells were harvested by centrifugation and re-suspended in phosphate-buffered saline (PBS), and then were broken by ultrasonication and the supernatant was isolated and purified by the GST-Tagged Protein Purification Kit (CWBIO, Beijing, China). Next, the purified RcCO/RcCOL4, RcNF-YB, and RcNF-YC proteins were mixed with probes (biotin-labeled and unlabeled) and incubated at 24 °C for 20 min, followed by separation on 6% native polyacrylamide gels in 0.5× TBE buffer. The gels were electroblotted to Hybond N+ (Millipore) nylon membranes in 0.5× TBE buffer for 210 min (120 mA) and then detected by Gel Documentation Systems (BIO-RAD Technology) (Xu *et al.*, 2014). The probes used in this study are listed in Supplementary Table S1.

### Pull-down assay

The pull-down assays were conducted using a HIS Pull-down kit (ThermoFisher Scientific) according to the manufacturer’s instruction. Briefly, the purified RcCO-His fusion protein was incubated with immobilized glutathione *S*-transferase (GST) and RcCOL4–GST fusion proteins in pull-down buffer (50 mmol l^–1^ Tris–HCl, pH 7.2, 150 mmol l^–1^ NaCl, 10% glycerol, 0.1% Triton X-100, 1× protease inhibitor cocktail) for 2 h at 4 °C. Then, proteins were eluted in the elution buffer, and the interaction was determined by western blot using anti-His antibody (CWBIO, Beijing, China).

### Statistical analyses

To determine statistical significance, these analyses were performed by Kruskal–Wallis test. The difference was considered significant at *P*<0.05.

## Results

### Converse regulation of *RcCO* and *RcCOL4* expression levels by photoperiod

**Fig. 1.**
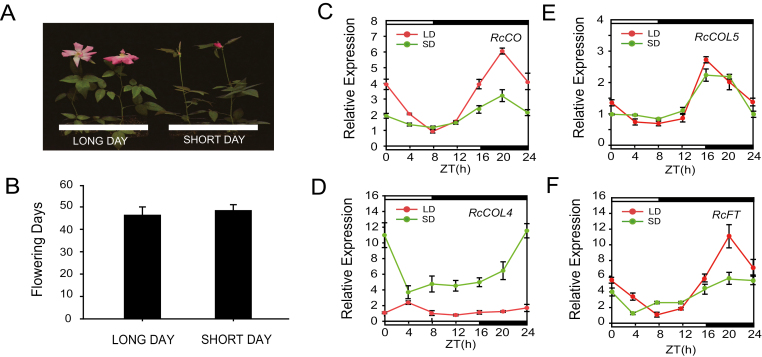
Flowering phenotype and expression levels of *RcCO*, *RcCOL4*, *RcCOL5*, and *RcFT* under LD and SD conditions. (A) Phenotypic characterization of *Rosa chinensis* ‘Old Blush’ under LD (16:8 h, light:dark) and SD (8:16 h, light:dark) conditions. (B) Flowering time of *R. chinensis* ‘Old Blush’ under LD and SD conditiona. Error bars indicate ± the standard deviation (*n*=10). (C–F) Relative expression of *RcCO* (C), *RcCOL4* (D), *RcCOL5* (E), and *RcFT* (F) of ‘Old Blush’ under LD and SD conditions. Error bars indicate ± the standard deviation (*n*=3).

CF roses are usually considered as DN plants because they flower under any favorable environments irrespective of the photoperiodic conditions. To confirm this, we compared the flowering time of CF cuttings of *R. chinensis* cv ‘Old Blush’ transplanted in LD and SD conditions. As shown in Fig. 1A and B, seedling cuttings bloomed at 43 d under LDs in contrast to 44 d under SDs. The difference in flowering time between SDs and LDs was not statistically significant, supporting the DN response of rose plants.

A conserved role of the *CO*/*FT* pathway in flowers of Arabidopsis and rice has been demonstrated, and the circadian regulation of *CO* as a basis for monitoring daylength and a guarantee for the induction of *FT* has been established (Suárez-López *et al.*, 2001; Valverde *et al.*, 2004). To clone *CO* in rose, we screened the newly published genome database of *R. chinensis* (https://lipm-browsers.toulouse.inra.fr/pub/RchiOBHm-V2/) (Raymond *et al.*, 2018), and identified 18 non-redundant BBX genes that contained one or two BBX domains (Supplementary Fig. S1). Three genes (*RcChr2g0164091*, *RcChr6g0299051*, and *RcChr4g0403841*) were classified into the structure group I subfamily, which contained a highly conserved double B-box domain in the N-terminus and a CCT domain in the C-terminus (Supplementary Fig. S2). Accordingly, the genes were designated as *RcCO* (*RcBBX1*), *RcCOL4* (*RcBBX5*), and *RcCOL5* (*RcBBX6*) following the nomenclature system suggested by Khanna *et al.* (2009).

Subsequently, the expression levels of *RcFT*, *RcCO*, and its closest family members *RcCOL4* and *RcCOL5* were examined every 4 h in a 24 h cycle starting at the onset of light. The results clearly showed circadian regulation of the genes, categorizing them according to the expression differences between SD and LD conditions: higher in LDs and lower in SDs (*RcCO*) (Fig. 1C), lower in LDs and higher in SDs (*RcCOL4*) (Fig. 1D), and lower or higher alternately (*RcFT* and *RcCOL5*) in SDs and LDs (Fig. 1E, F). It was noteworthy that the higher expression of *RcFT* from ZT8 to ZT16 may compensate the lower expression in the rest time under SDs, which may result in the equivalent flowering time in SDs and LDs (Fig. 1F). Interestingly, *RcCO* and *RcCOL4* displayed distinct photoperiod-dependent expression levels; that is, the expression level of *RcCOL4* was higher in SDs than in LDs, whereas the level of *RcCO* was higher in LDs than SDs over most times of the day and light cycle. To further test the universality of this phenomenon, three OF (*R. laevigata*, *R. berberifolia*, and *R. multiflora*) and CF (*R. chinensis* cv ‘Sichun’, *R. chinensis* cv ‘Viridiflora’, and *R. hybrida* cv ‘Molde’) rose varieties were selected to characterize the time-course of *RcCO*, *RcCOL4*, and *RcCOL5* expression in a 24 h cycle under both LDs and SDs. Surprisingly, *RcCOL4* was expressed preferentially more highly under SDs in all the CF varieties (Supplementary Fig. S3B), in contrast to the higher expression levels of *RcCO* under LDs, in both OF and CF rose varieties (Supplementary Fig. S3A), while there was no obvious regularity of *RcCOL4* expression under LDs in OF roses (Supplementary Fig. S3B) and of *RcCOL5* in OF and CF roses (Supplementary Fig. S3C). Collectively, these results portrayed inverse responses of *RcCO* and *RcCOL4* expression to the photoperiod and thus suggested their key roles in photoperiod-dependent flowering time.

### *RcCO* and *RcCOL4* are essential for DN response of roses

**Fig. 2.**
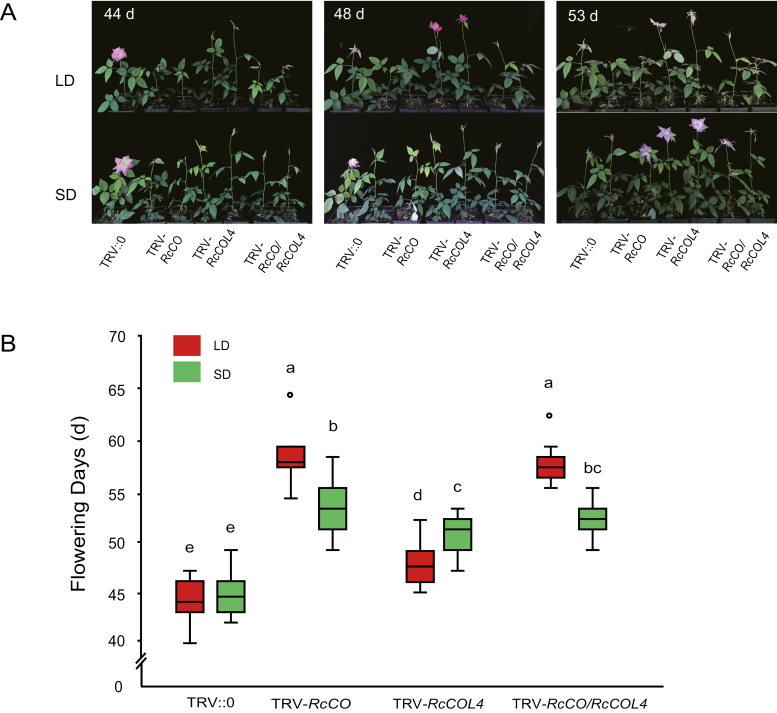
Flowering phenotype of TRV-*RcCO* and TRV-*RcCOL4* plants under LD and SD conditions. (A) Time-course of the flowering phenotype of TRV-*RcCO* and TRV-*RcCOL4 Rosa chinensis* ‘Old Blush’ under LD and SD conditions. (B) Flowering time of TRV-silenced *R. chinensis* ‘Old Blush’ under LD and SD conditions. Error bars indicate ± the standard deviation (*n*=10). Different letters above the columns denote significant differences as determined by Kruskal–Wallis test (*P*＜0.05).

**Fig. 3.**
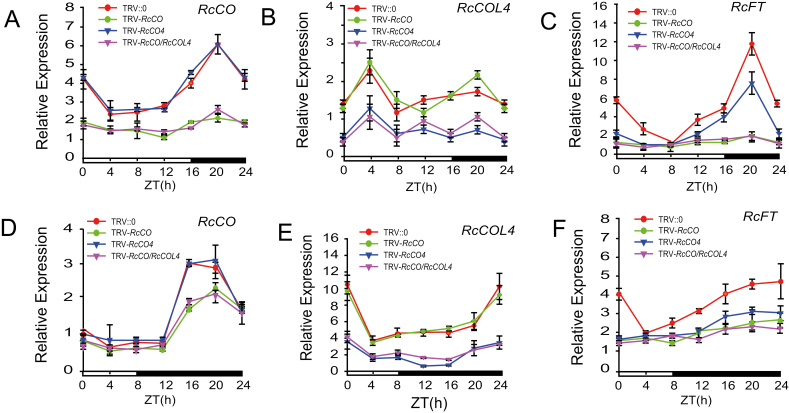
Time-course expression levels of *RcCO*, *RcCOL4*, and *RcFT* in TRV-*RcCO* and TRV-*RcCOL4* plants under LD and SD conditions. (A–C) Relative expression of *RcCO* (A), *RcCOL4* (B), and *RcFT* (C) in TRV-silenced *Rosa chinensis* ‘Old Blush’ under LDs. Error bars indicate ± the standard deviation (*n*=3). (D–F) Relative expression of *RcCO* (D), *RcCOL4* (E), and *RcFT* (F) in TRV-silenced *R. chinensis* ‘Old Blush’ under SDs. Error bars indicate ± the standard deviation (*n*=3). The uppermost young leaves from 40-day-old plants propagated from cuttings were harvested and the total RNAs were extracted. RT-qPCR was then performed with *RcGAPDH* as reference gene. Every experiment was conducted with three replicates each with three technical repeats. The bar below the graphs indicates the light conditions, with day and night denoted in white and black, respectively.

To gain the genetic evidence of the biological functions of *RcCO*, *RcCOL4,* and *RcCOL5*, the VIGS technique was employed to silence the genes in *R. chinensis* ‘Old Blush’, followed by recoding their respective flower phenotypes (Fig. 2; Supplementary Fig. S4), and measurements of gene expression under SD and LD conditions (Fig. 3, Supplementary Fig. S4). *RcFT* was also quantified as a marker gene of flowering time (Fig. 3C, F). As shown in Fig. 2A, B and Supplementary Fig. S4, the flowering times of *RcCO*-, *RcCOL4*-, and *RcCOL5-*silenced plants differed under both LD and SD conditions with the decrease of gene expression. Specifically, flowering of *RcCO*-silenced plants was delayed 14 d in LDs and 9 d in SDs, flowering of *RcCOL4*-silenced plants was delayed 4 d in LDs and 7 d in SDs, whereas flowering of *RcCOL5*-silenced plants was delayed 6 d in LDs and SDs. Consequently, *RcCO*-silenced plants flowered later in LDs (57 d) than in SDs (53 d), *RcCOL4*-silenced plants flowered earlier in LDs (47 d) than SDs (51 d), while *RcCOL5*-silenced plants flowered at the same time (50 d) under LDs and SDs. These results clearly indicated that *RcCO*, *RcCOL4*, and *RcCOL5* were all flowering activators in rose plants and essential for normal flowering under SD and LD conditions. It was noteworthy that only silencing of either *RcCO* or *RcCOL4* induced the difference in flowering time between SDs and LDs, thus disturbing the DN response of rose plants, ruling out the function of *RcCOL5* in the DN response. Furthermore, the identical flowering time between *RcCO/RcCOL4* double-silenced plants and *RcCO*-silenced single mutants implied the epistatic function of *RcCOL4* and *RcCO* (Fig. 2A, B).

As the key flowering integrator, the expression levels of *RcFT* were reduced significantly under both LDs and SDs in *RcCO*- and *RcCOL4*-silenced plants (Fig. 3C, F), consistent with the aforementioned flowering phenotypes (Fig. 2A, B). Specifically, the expression of *RcFT* in *RcCO*-silenced plants was higher in SDs than in LDs; in contrast, that in *RcCOL4*-silenced plants was lower in SDs than in LDs. This implied that an imbalance of *RcFT* expression could by extension compromise the DN response. These results suggested that *RcCO* and *RcCOL4* regulate flowering time through affecting the transcription of *RcFT*, and that the complementary expression of *RcCO* in LDs and of *RcCOL4* in SDs guarantees rose flowering under favorable conditions irrespective of the photoperiod, and facilitation of the DN responses of CF roses.

### *RcCO* rather than *RcCOL4* recovers the late flowering phenotype of the *co* mutant in Arabidopsis

**Fig. 4.**
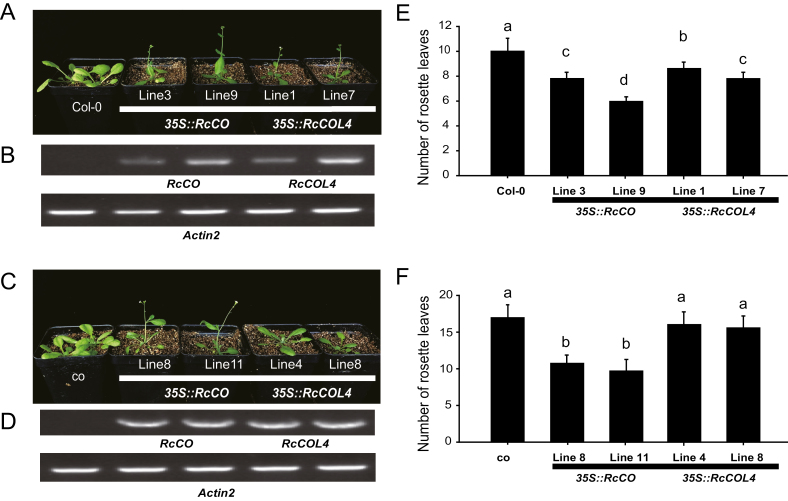
Flowering phenotype of *RcCO-* and *RcCOL4*-overexpressing Arabidopsis in the Col and *co* background. (A and E) Flowering phenotypes and rosette leaf numbers of *RcCO*- and *RcCOL4*-overexpressing plants in the Col background. (B and D) Expression of *RcCO* and *RcCOL4* measured by semi-quantitative RT-PCR in Arabidopsis. *Actin2* was used as reference gene. (C and F) Flowering phenotypes and rosette leaf numbers of *RcCO*- and *RcCOL4*-overexpressing plants in the *co* mutant background. Error bars indicate ± the standard deviation (*n*=3). Different letters above the columns denote significant differences at *P<*0.05.

To further test the functions of *RcCO* and *RcCOL4* in Arabidopsis, we overexpressed them in Col wild type (WT) as well as in the *co* mutant background. We performed semi-quantitative RT-PCR in Arabidopsis to verify the expression of *RcCO* and *RcCOL4* (Fig. 4B, D). Based on the rosette leaf number under LD conditions, the flowering time of overexpression lines was accelerated significantly in WT backgrounds. For example, the number of rosette leaves of *RcCO* or *RcCOL4* overexpression lines at flowering initiation was seven or eight, respectively, in contrast to 10 leaves in the WT (Fig. 4A, E). In terms of the *co* mutant, the flowering time was delayed significantly to 18 rosette leaves in comparison with 10 in the WT, overexpression of *RcCO* decreased the number of rosette leaves to nine, while no obvious phenotype was observed upon overexpression of *RcCOL4* in the *co* mutant background (Fig. 4C, F). In summary, the complementary effect on flowering time of *co* mutants by *RcCO* demonstrated the conserved function of *RcCO* and *AtCO* in flowering regulation. Furthermore, *AtCO* most probably acted downstream of *RcCOL4* and was essential for its function in Arabidopsis, consistent with the previous result in roses.

### RcCO regulates *RcFT* by directly binding to the CORE motif in the promoter

**Fig. 5.**
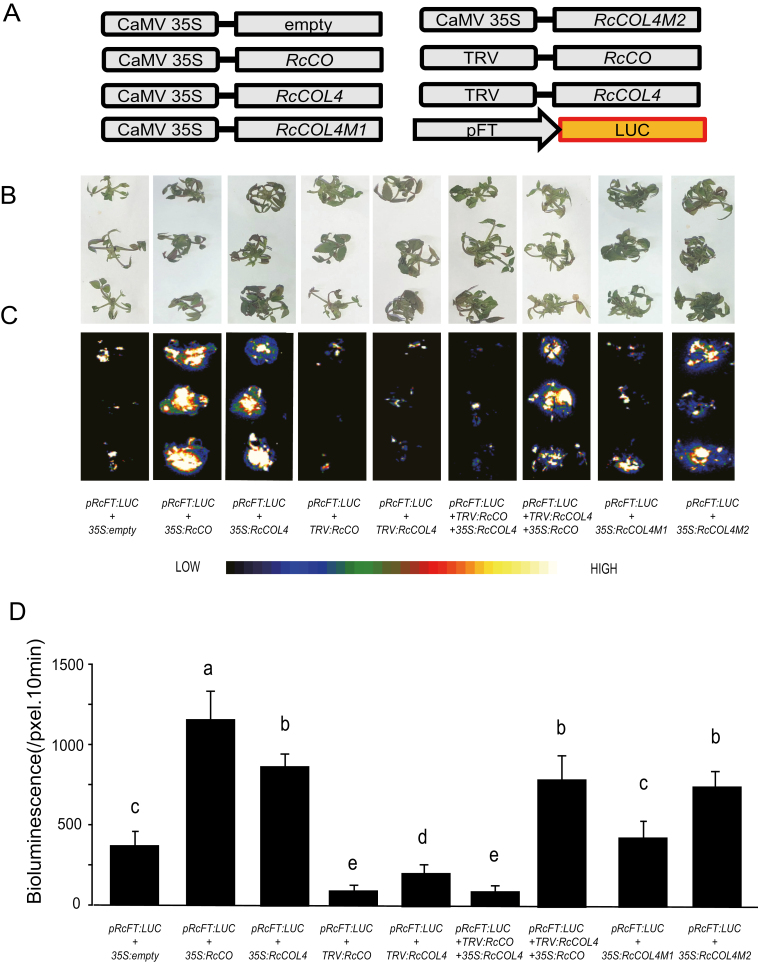
Transient transformation analysis of transcriptional activation of *RcFT* by *RcCO* and *RcCOL4.* (A) Schematic diagram of the reporter and effectors used in the *Rosa chinensi*s ‘Old Blush’ transient transformation assays. (B and C) Representative images of transient expression assays in *R. chinensis* ‘Old Blush’ displayed by bright field (B) and dark field (C) of rose shoots expressing *pRcFT-LUC* together with 35S:empty, 35S:*RcCO*, 35S:*RcCOL4*, TRV-*CO*, TRV-*COL4*, TRV-*CO*+35S:*RcCOL4*, TRV-*COL4+* 35S:*RcCO*, mutated *RcCOL4-M1*, and *RcCOL4-M2*. (D) Intensities of the LUC bioluminescence presented in (C) using Andor Solis image analysis software. Data are means ± SE (*n*=10). Different letters above the columns denote significant differences at *P*<0.05.

In Arabidopsis, *AtCO* activates *AtFT* transcription through direct binding to CO-responsive (CORE) elements in the *FT* promoter (Seung Kwan *et al.*, 2005; Wenkel *et al.*, 2006; Tiwari *et al.*, 2010; Cao *et al.*, 2014). This prompted us to test the relationship between *RcCO* and *RcFT* in roses. We performed transient *A. tumefaciens* infiltration assays in the young rose shoots as previously described (Lu *et al.*, 2017). Specifically, a construct containing the *FT* promoter region fused to firefly luciferase (*pRcFT:LUC*) was infiltrated into ‘Old Blush’ young shoots together with an empty vector control, or in *35S:RcCO* or *35S:RcCOL4* (Fig. 5A). The results clearly demonstrated a notable induction above the LUC bioluminescence background in rose shoots co-infiltrated with *pRcFT:LUC* plus *35S:RcCO* or *35S:RcCOL4* (Figs. 5B–D), suggesting that RcCO and RcCOL4 might activate the expression of *RcFT*. Consistently, the base level of LUC bioluminescence reflecting the promoter activity of *RcFT* was suppressed significantly by silencing of *RcCO* or *RcCOL4*. Interestingly, the promotion effect of RcCOL4 on *pRcFT:LUC* was almost eliminated in *RcCO*-silenced seedlings, while enhanced *pRcFT:LUC* activity by RcCO was not affected in *RcCOL4*-silenced seedlings. Collectively these results suggested that RcCO and RcCOL4 activate the expression of *RcFT*, thus accelerating flowering time, and furthermore with a functional RcCOL4 depending on RcCO. Subsequently, the binding of RcCO to the *RcFT* promoter was further confirmed in yeast one-hybrid assays; however, RcCOL4 was not shown to directly bind to the promoter of *RcFT* in yeast cells (Supplementary Fig. S5).

**Fig. 6.**
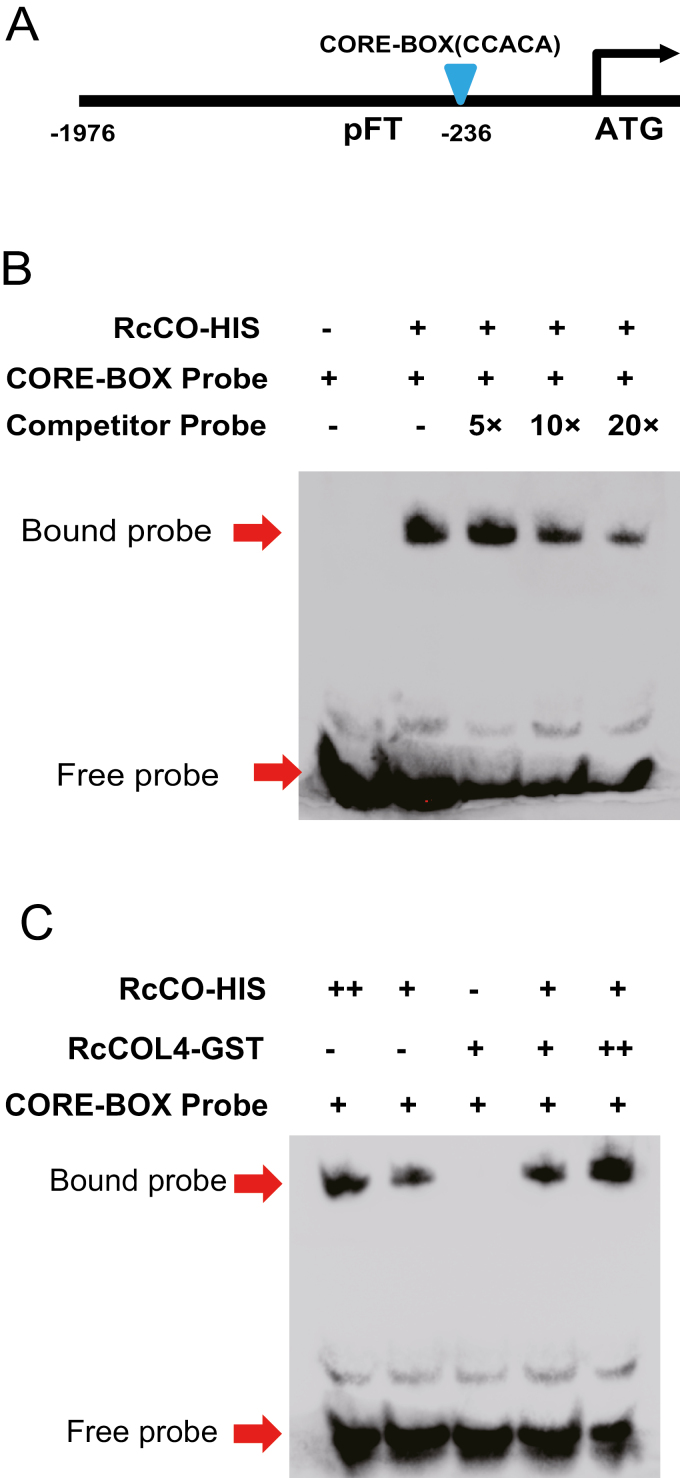
*RcCOL4* facilitates the binding of *RcCO* to the CORE motif in the promoter of *RcFT*. (A) Location of the CORE motif in the promoter of *RcFT*. (B) Direct binding of RcCO to the CORE motif of the *RcFT* promoter in *in vitro* EMSA. Biotin-labeled probes were incubated with RcCO-HIS protein, and the free and bound probes were separated on an acrylamide gel. 5×, 10×, and 20× represent the dilution multiples of the competitor probe. (C) RcCOL4 enhances the binding ability of RcCO to the CORE motif in the promoter of *RcFT*. Biotin-labeled probes were incubated with RcCO-HIS alone or together with RcCOL4–GST protein, and the free and bound probes were separated on an acrylamide gel.

To further clarify the binding details, we screened the promoter of *RcFT* and found the CORE motif located at –236 bp upstream of the start codon (Fig. 6). Next we generated biotinylated probes across the motif for EMSA. The EMSA result (Fig. 6B) clearly demonstrated RcCO direct binding to the CORE motif in the *RcFT* promoter as determined by mobility shift, and the binding activities decreased dose-dependently by competitive probes. To further differentiate the function of *RcCO* and *RcCOL4* using the EMSA system, RcCO and RcCOL4 proteins were incubated with the labeled probes in different combinations. The results clearly showed that only RcCO and not RcCOL4 bound to the CORE motif in the promoter of *RcFT*; however, the combination of RcCO with RcCOL4 certainly enhanced the binding activity (Fig. 6C).

Collectively, these results from the transient binding analysis, yeast one-hybrid assay, and EMSA provided solid evidences that RcCO directly bound to the promoter of *RcFT* via the CORE motif to activate its expression, and that binding was enhanced by RcCOL4, suggesting that RcCO is the downstream target of RcCOL4 that is indispensable for a functional RcCOL4.

### The Box1 motif of RcCOL4 is indispensable for the RcCO–RcCOL4 interaction

**Fig. 7.**
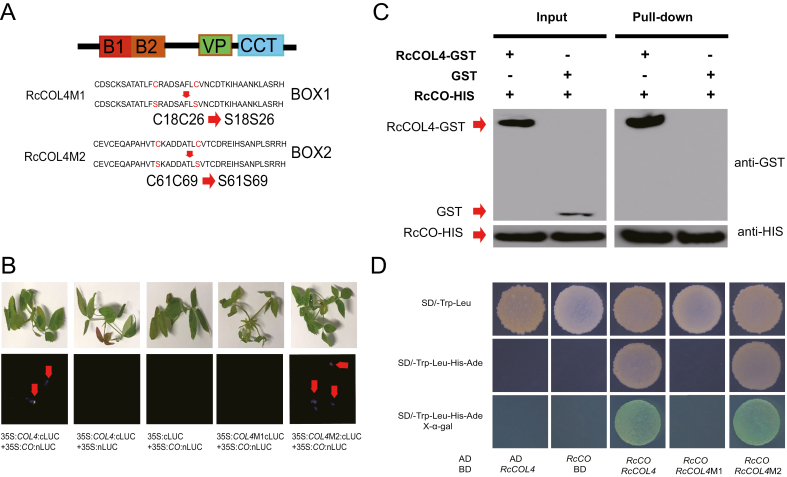
The Box1 motif of *RcCOL4* is indispensable for the RcCO–RcCOL4 protein interaction. (A) Schematic representation of the amino acid sequences and mutations of the B-box motif in *RcCOL4*. (B) Representative images of the spilt luciferase complementation assays in *Rosa chinensis* ‘Old Blush’ displayed by bright field and dark field of rose seedlings co-expressing *RcCO* and *RcCOL4*, and *RcCO* and mutated *RcCOL4*. (C) Pull-down assays prove the interaction between RcCOL4 and RcCO. The purified RcCO-HIS fusion protein was incubated with immobilized GST and RcCOL4–GST fusion proteins in pull-down buffer and the interaction was determined by western blot. (D) Yeast two-hybrid assay verifies the interaction between RcCOL4 and RcCO, and of RcCOL4-M2, but not RcCOL4-M1, and RcCO.

Given that RcCOL4 enhanced binding of RcCO to the promoter of *RcFT*, we questioned whether RcCOL4 could physically interact with RcCO. Thus, we conducted split luciferase complementation assays by fusing RcCOL4 and RcCO to the N- or C-terminal fragments of luciferase, respectively. Subsequently, these constructs were used in agroinfiltration-based transient assays in ‘Old Blush’ seedlings as we previously described (Lu *et al.*, 2017). The outcome clearly revealed reconstitution of luciferase activity in the rose shoots co-infiltrated with RcCOL4–cLUC and RcCO–nLUC (Fig. 7B), thus, showing physical interaction between RcCOL4 and RcCO. Moreover, the interaction results were also verified using a pull-down assay and a Y2H assay (Fig. 7C, D).

Next, we performed luciferase activity reconstitution assays with two independently mutated *RcCOL4* constructs to better understand the role of individual B-boxes in the RcCOL4–RcCO protein interaction. One mutant construct contained Cys18 and Cys26 substituted to Ser in Box1 (named RcCOL4M1) and the other contained the analogous substitution Cys61 and Cys69 to Ser in Box2 (named RcCOL4M2) (Fig. 7A). These data clearly showed that the interaction between RcCOL4 and RcCO was eliminated in rose shoots co-infiltrated with RcCO and RcCOL4M1, whereas the protein–protein interaction remained unaffected by mutation in Box2 (Fig. 7B). These results were verified using a yeast hybrid assay (Fig. 7D).

Because of the critical role of Box1 in *RcCOL4* for protein interaction, we further investigated whether the mutated *RcCOL4* still possesses the ability to regulate *FT* transcription. Thus, we performed transient assays in rose seedlings using *pFT:LUC* plus empty vector control, *35S*:*RcCOL4*, *35S*:*RcCOL4-M1*, or *35S*:*RcCOL4-M2*. Comparing the LUC bioluminescence in the rose shoots, the enhancement of LUC activity by *35S:RcCOL4* was almost abolished in *35S*:*RcCOL4-M1* but not in *35S*:*RcCOL4-M2* (Fig. 5B, C). This finding further defined the indispensable role of Box1 for the function of *RcCOL4* in *RcFT* promotion and flowering time regulation in *R. chinensis*.

## Discussion

The timing of flowering is an essential determinant for the adaptation to different environments by plants. The transition from vegetative to reproductive development is triggered by a leaf-derived, mobile floral-promoting signal named florigen (Chailakhyan, 1937; Giakountis and Coupland, 2008). The florigen-encoding *FT* genes are extensively identified and their functions are conserved among SD, LD, and DN plants. Conditional accumulations of FT to the threshold required for flowering are critical and common to all three types of photoperiodic response plants. Orthologs of the *FT* gene accelerate flowering in the LD Arabidopsis and the SD rice. The universal florigenic signal triggered by *FT* homologs is known to regulate growth and flowering cycles in perennial DN tomato (*S. lycopersicum*) (Lifschitz and Eshed, 2006). In maize (*Z. mays*), the florigen gene *ZCN8* is associated with the floral transition in both DN temperate maize and SD-requiring tropical maize, and has been shown to be regulated by different chromatin modifications at the floral transition (Lazakis *et al.*, 2011).

The mechanisms underlying plant photoperiodic responses can be explained by the external coincidence model. In this model, the coincidence of a photoperiodic signal perceived by photoreceptors and internal gene expression during a specific phase determines flowering (Searle and Coupland, 2014). CO is a transcription factor which acts as a time keeper. In Arabidopsis, the circadian regulation of *CO* transcript levels in conjunction with the light-induced stabilization of CO protein peaking at dusk is an established basis for monitoring daylength and a guarantee for the induction of *FT* under LDs (Suárez-López *et al.*, 2001; Valverde *et al.*, 2004). The CO–FT module also controls photoperiodic flowering in rice and poplar, but in the SD plant rice, *Hd3a* (the homolog of Arabidopsis *FT* in rice) is induced when the *CO* homolog *Hd1* peaks during the night (Shoko *et al.*, 2002; Henrik *et al.*, 2006). The reverse response to daylength observed between Arabidopsis (LD plant) and rice (SD plant) is partly explained by the difference in the function of *CO* in Arabidopsis and the rice homolog *Hd1* (Izawa *et al.*, 2002; Ryosuke *et al.*, 2003). However, the DN response is the most poorly characterized among the three types of photoperiodic flowering responses.

Although there are three different flowering modes (OF, CF, and OB) in rose plants, the CF trait is much more popular and plays an essential role in the tremendous success of modern roses. In contrast to OF, CF (also called recurrent, perpetual, everbearing, or remontant flowering) rose varieties start to flower in spring and continuously initiate new flowers until late autumn (Sønsteby and Heide, 2007; Foucher *et al.*, 2008), and hence are generally considered as DN plants (Zieslin and Moe, 1985). In the present study, normal flower initiation in CF rose *R. chinensis* ‘Old Blush’ occurred under both SD and LD conditions, supporting roses as DN plants. In line with the flowering phenotype, the expression level of *RcFT* was higher under LDs and SDs alternately in the day and night cycle. Additionally, the CO–FT module in *R. chinensis* was also conserved in flowering time regulation, which was in agreement with the published literature in other species (Izawa *et al.*, 2002; Ryosuke *et al.*, 2003). *RcCO* was expressed more under LD conditions and directly bound to the CORE motif of the *RcFT* promoter to activate its expression (Figs 5, 6); accordingly, silencing of *RcCO* in *R. chinensis* by VIGS delayed flowering time significantly under both SDs and LDs (Fig. 2). Interestingly, *RcCOL4*, a close member of subgroup I of the BBX gene family, showed higher expression under SDs in the CF rose and physically interacted with RcCO to enhance its binding to the promoter of *RcFT* (Figs 1, 6, 7). As a result, flowering time of *RcCOL4*-silenced plants was later in SDs than in LDs. In contrast, *RcCO*-silenced plants flowered earlier in SDs than in LDs upon perturbation of the DN response, implying the important role of *RcCOL4* under SDs and *RcCO* under LDs in rose flowering. Collectively, these data suggested that the alternate expression of *RcCOL4* in SDs and of *RcCO* in LDs facilitates the CF trait and DN response of *R. chinensis*.

In Arabidopsis, control of flowering time is not limited to *CO*/*BBX1*, since other BBX family members also regulate flowering through distinct and overlapping as well as antagonistic functions (Cheng and Wang, 2005; Datta *et al.*, 2006; Hassidim *et al.*, 2009; Park *et al.*, 2011; Li *et al.*, 2014). In contrast, the function of BBX family members has never been characterized in rose plants. Here, we identified three BBX genes, *RcCO*, *RcCOL4*, and *RcCOL5*, as flowering accelerators in *R. chinensis*. We further showed that three BBXs were required for rose normal flowering under LD and SD conditions. Silencing either of *RcCO*, *RcCOL4*, or *RcCOL5* delayed flowering under both LDs and SDs; however, *RcCOL4*-silenced plants flowered later in SDs than in LDs, in contrast to *RcCO*-silenced plants which flowered later in LDs than in SDs. The results established the distinct roles of RcCO and RcCOL4 in response to different photoperiods and they coordinately enabled the DN response of *R. chinensis*. A previous study showed that the CF phenotype of roses was mainly caused by a dysfunctional flowering repressor *KSN*, a *TFL1* homolog of Arabidopsis, which was specifically inhibited by SDs and activated by LDs in *Fragaria vesca* (Koskela *et al.*, 2012). The identification of *RcCOL4* as a floral promoter preferably in SDs provided a new angle to better understand the mechanism of the CF trait, supporting the notion that the CF trait may be controlled by multiple regulators.

**Fig. 8.**
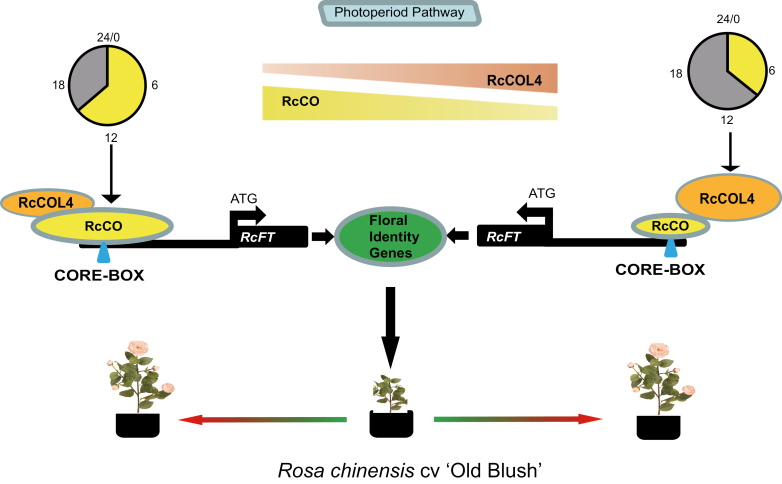
Simplified schematic model of flowering time regulation by *RcCOL4*–*RcCO* in *Rose chinensis* under different daylengths. Under LD conditions, *RcCO* is expressed more highly and plays a prominent role in flowering promotion via direct binding to the promoter of *RcFT* to activate its expression; under SD conditions, *RcCO* is down-regulated while *RcCOL4* increases and accelerates flowering via physically interacting with *RcCO* to enhance its binding to *FT*. Consequently, *R. chinensis* ‘Old Blush’ could flower under both LDs and SDs.

In conclusion, the present results lead to the proposal of a new model for the continuous flowering in CF cultivar *R. chinensis* ‘Old Blush’ (Fig. 8). Under LD conditions, *RcCO* was more highly expressed and played a prominent role in flowering promotion via direct binding to the promoter of *RcFT* to activate its expression. Under SD conditions, *RcCO* expression levels were reduced, but *RcCOL4* enabled accelerated flowering via physically interacting with *RcCO* to enhance its binding to *RcFT*. Consequently, *R. chinensis* could continuously flower under both SDs and LDs irrespective of the photoperiodic conditions.

## Supplementary data

Supplementary data are available at *JXB* online.

Fig. S1. Identification of *RcBBX* family genes in *Rosa chinensis*.

Fig. S2. Amino acid sequence comparison of CO, COL4, and COL5 across different species

Fig. S3. Expression level of *CO*, *COL4*, and *COL5* in three OF and three CF roses under LD and SD conditions.

Fig. S4. Flowering phenotype and gene expression levels of *RcCOL5*-silenced plants under LDs and SDs.

Fig. S5. *RcCO* not *RcCOL4* binds to the promoter of *RcFT* in yeast one-hybrid assay.

Table S1. Primers used in this study,

eraa161_suppl_Supplementary_Figures_S1-S5_Table_S1
