# Supplementary material for: Alternate expression of CONSTANS-LIKE 4 in short days and CONSTANS in long days facilitates day-neutral response in Rosa chinensis
Source: J Exp Bot. 2020 Mar 28;71(14):4057–68. doi: 10.1093/jxb/eraa161 (PMC7475255; doi:10.1093/jxb/eraa161)
Supplement: eraa161_suppl_Supplementary_Figures_S1-S5_Table_S1 [file eraa161_suppl_supplementary_figures_s1-s5_table_s1.pdf]

A

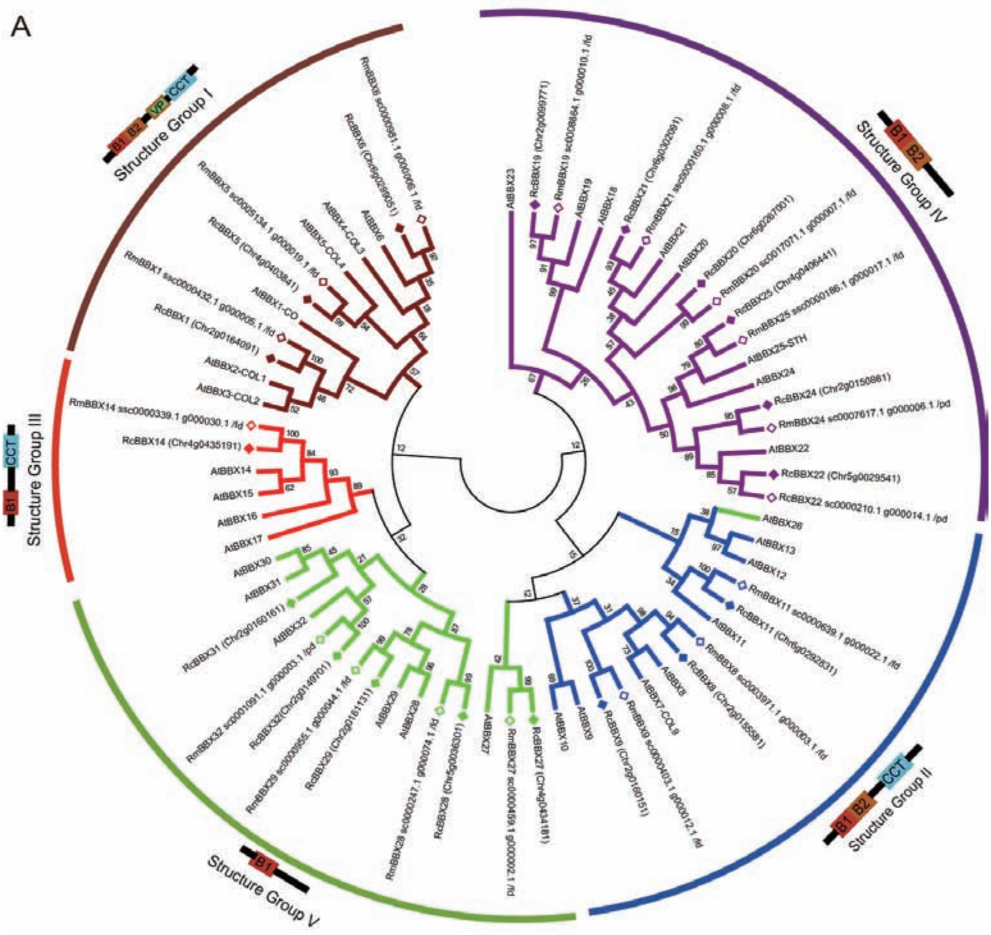

B

Species	All	Group I	Group II	Group III	Group IV	Group V	Chromosome
Arabidopsis thaliana	32	6	7	4	8	7	2n=10
Rosa chinensis	18	3	3	1	6	5	2n=14
Rosa multiflora	17	3	3	1	6	4	2n=14

Figure S1. Identification of *RcBBX* family genes in *Rosa chinensis*

(A) Phylogenetic analysis of BBX family genes among Arabidopsis thaliana, *Rosa multiflora* and *Rosa chinensis*. Bootstrap values indicate the divergence of each branch. Different groups are classified and shown by different color.

(B) Numbers of BBX family genes among Arabidopsis thaliana, *Rosa multiflora* and *Rosa chinensis*.

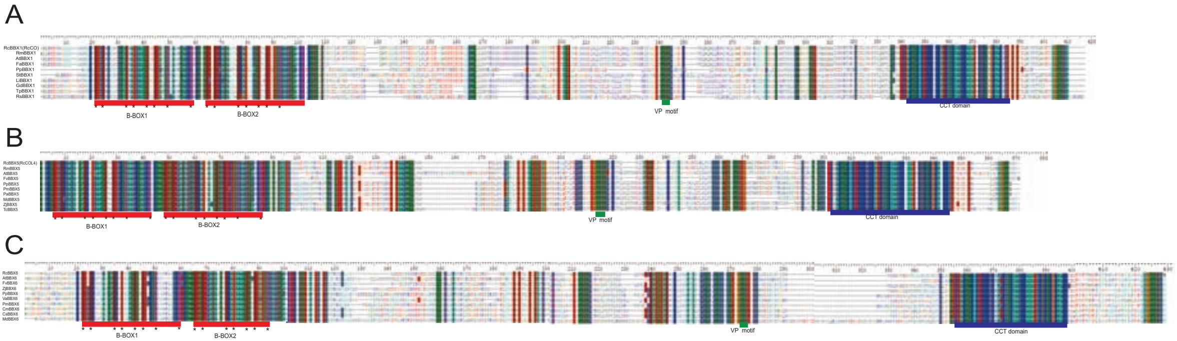

Figure S2. Amino acid sequence comparison of *CO*, *COL4* and *COL5* across different species

Alignment of amino acid sequences of *RcCO* **(A)**, *RcCOL4* **(B)** and *RcCOL5* **(C)** with the counterparts from *Rosa chinensis* (Rc), *Rosa multiflora* (Rm), *Arabidopsis thaliana* (At), *Fragaria* × *ananassa* (Fa), *Prunus persica* (Pp), *Solanum tuberosum* (St), *Lagerstroemia indica* (Li), *Gossypium darwinii* (Gd), *Thespesia populneoides* (Tp), *Raphanus sativus* (Rs), *Fragaria vesca* (Fv), *Prunus avium* (Pa), *Ziziphus jujube* (Zj), *Theobroma cacao* (Tc), *Vigna angularis* (Va), *Prunus mume* (Pm), *Cucumis sativus* (Cs), *Cucumis melo* (Cm), *Malus domestica* (Md). Red lines indicate two B-box domains, green lines indicate VP motif and blue lines indicate CCT domain. The asterisks below the red lines indicate the Zn^{2+}-ligating conserved Cys, His and Asp residues presented in the B-box.

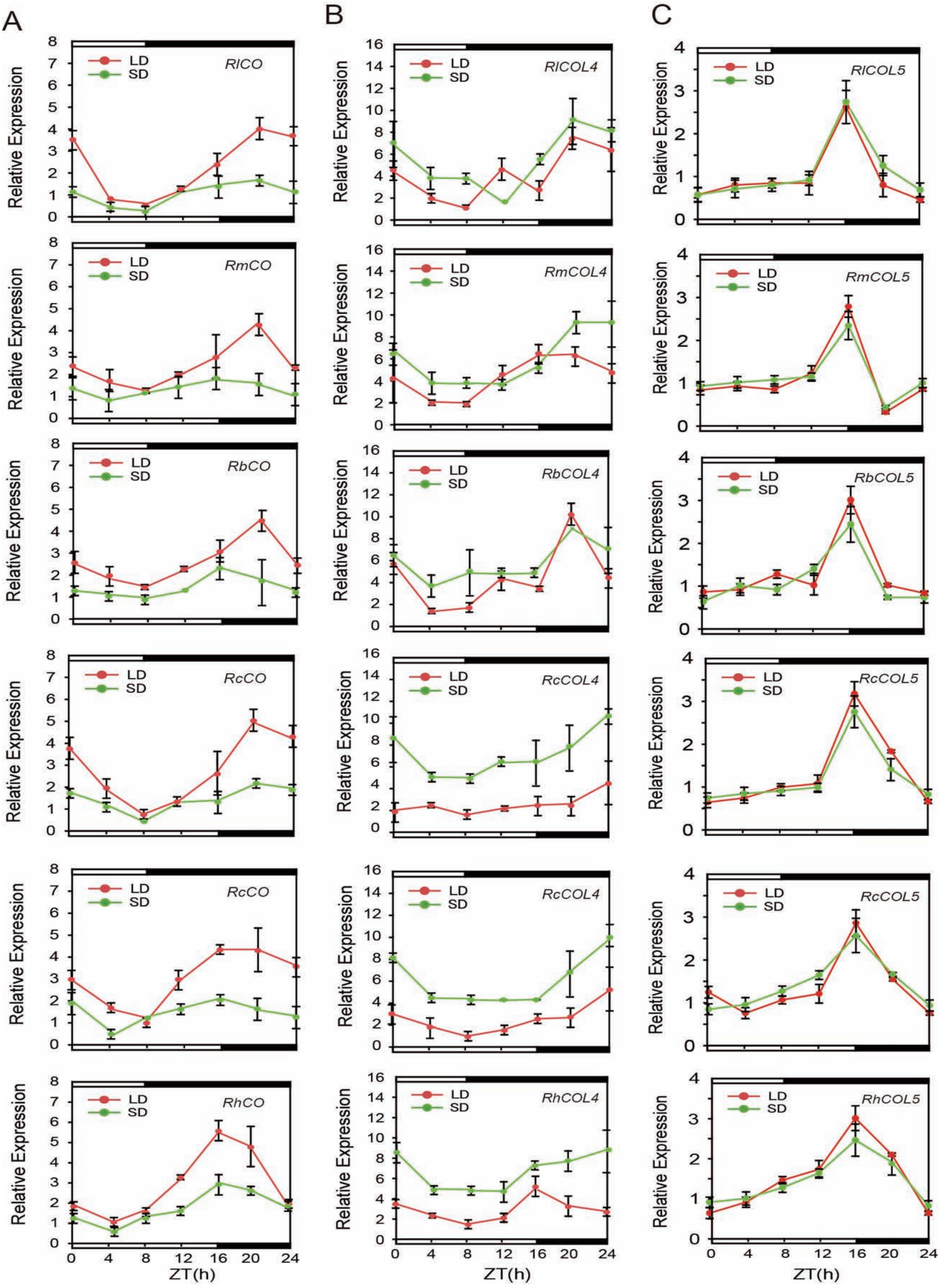

Figure S3. Expression level of *CO*, *COL4* and *COL5* in three OF and three CF roses under LD and SD condition.

(A) Relative expression of *CO* in three OF roses (*Rosa laevigata*, *Rosa multiflora* and *Rosa berberifolia*) and three CF roses (*Rosa chinensis* cv 'Sichun', *Rosa chinensis* cv 'Viridiflora' and *Rosa hybrida* cv 'Molde').

(B) Relative expression of *COL4* in three OF roses (*Rosa laevigata*, *Rosa multiflora* and *Rosa berberifolia*) and three CF roses (*Rosa chinensis* cv 'Sichun', *Rosa chinensis* cv 'Viridiflora' and *Rosa hybrida* cv 'Molde').

(C) Relative expression of *COL5* in three OF roses (*Rosa laevigata*, *Rosa multiflora* and *Rosa berberifolia*) and three CF roses (*Rosa chinensis* cv 'Sichun', *Rosa chinensis* cv 'Viridiflora' and *Rosa hybrida* cv 'Molde').

The bar above and below the graphs indicates the light conditions, with day and night denoted in white and black, respectively.

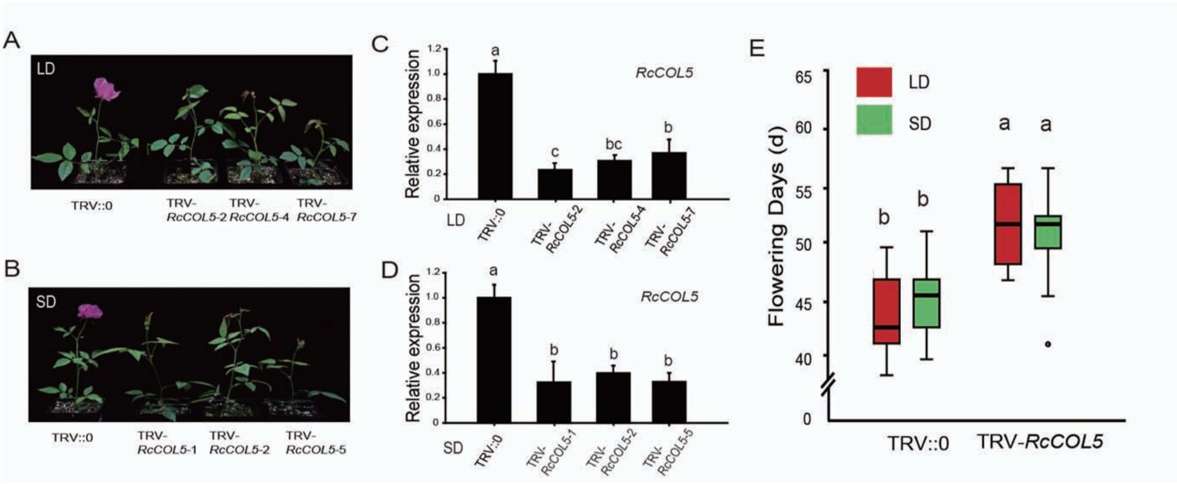

Figure S4. Flowering phenotype and gene expression levels of *RcCOL5*-silenced plants under LD and SD

(A), (B) Phenotypic characterization of TRV-*RcCOL5* plants under long day LD (A) and SD (B) conditions (Plants were photographed 44 days after transplanting).

(C), (D) Relative expression of *RcCOL5* in silenced plants under long day LD (C) and SD (D) conditions Error bars indicate ±SD (n=10). Letters above the bars denote significant differences ($P < 0.05$) .

(E) Flowering time of *RcCOL5*-silenced plants under LD and SD conditions. Error bars indicate ±SD (n=10). Letters above the bars denote significant differences as determined Kruskal Wallis test ($P <$ 0.05) .

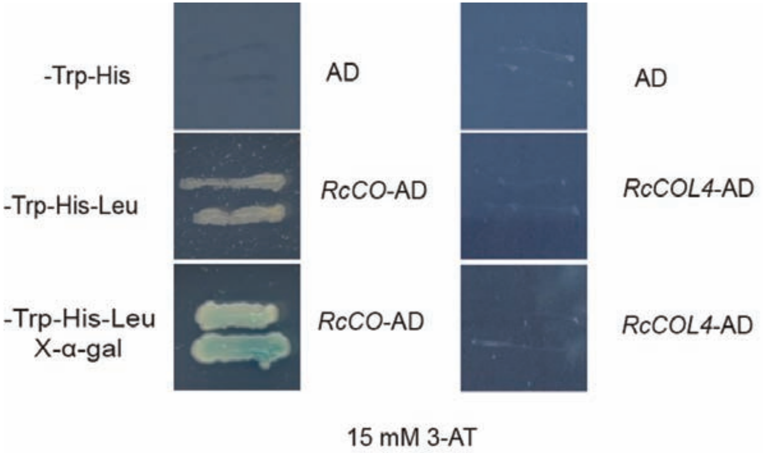

Figure S5. *RcCO* not *RcCOL4* binds to the promoter of *pRcFT* in yeast one hybrid.

Yeast Y187 cells containing pGADT7-*RcCO*/pGADT7-*RcCOL4* and pHis2-*pFT* were incubated, and the binding activities were examined on SD medium lacking Tryptophan, Leucine and Histidine (SD/−Trp/−Leu/−His) with the proper concentration of 3-amino-1, 2, 4-triazole (15mM) to inhibit self-activation.

Table S1. The primers used in this study.

Primer ID	Sequence of primers (5'-3')	Purpose
RcCO-F	caccATGTTGAAAGAAGAGAGCAATG	Gene cloning to D-TOPO
RcCO-R	GTATGAAGGAACAATGCCGTATC	
RcCOL4-F	caccATGTTTCACTCGGGGCAATC	Gene cloning to D-TOPO
RcCOL4-R	AAAAGAGGGAACGACGCCGTAGC	
pRcFT-F	caccATATCAGTTCTTCATGGCAATCAG	Promoter cloning to D-TOPO
pRcFT-R	TAACTAATTTTACACAGGCCACCT	
RcCO-F-EcoR I	GGAATTCATGTTGAAAGAAGAGAGCA	For yeast one/two hybrid
RcCO-R-Xho I	CCTCGAGGTATGAAGGAACAATGCCGTAT	
RcCOL4-F-Nde I	CCATATGATGTTTCACTCGGGGCAATC	For yeast one/two hybrid
RcCOL4-R-Sal I	GGTCGACAAAAGAGGGAACGACGCC	
pFT-pHis-F	GGAATTCATATCAGTTCTTCATGGCAATCAG	For yeast one hybrid
pFT-pHis-R	GGAGCTCTAACTAATTTTACACAGGCCACCT	
RcCO-F-BamH I	GGAATTCATGTTGAAAGAAGAGAGCA	Vectors for in vitro expression of protein
RcCO-R-Xho I	CCTCGAGGTATGAAGGAACAATGCCGTAT	
RcCOL4-F-BamH I	GGAATTCATGTTTCACTCGGGGCAATC	Vectors for in vitro expression of protein
RcCOL4-R-Xho I	CCTCGAGAAAAGAGGGAACGACGCCGTAGC	
NF-YB-F-BamH I	GGAATTCATGGCCGACTCGGACAACGACTC	Vectors for in vitro expression of protein
NF-YB-R-Xho I	CCTCGAGCCTTGACCTCACTGAAGCT	
NF-YC-F-BamH I	GGAATTCATGGATCAGCAAGGACATGGGCA	Vectors for in vitro expression of protein
NF-YC-R-Xho I	CCTCGAGATGATCAGATGGTGACTGTTGCTG	
RcCOL4-mu1-F	CTTTCCTCTCCGTAAACTGCGACACAAAGATCCACGC	For mutation of Cys-18
RcCOL4-mu1-R	CAGTTTACGGAGAGGAAAGCGGAGTCTGCTCGGCA	
RcCOL4-mu2-F	CGACTCTGTTCTCCCGAGCAGACTCCGCTTTCCTCT	For mutation of Cys-26
RcCOL4-mu2-R	TGCTCGGGAGAACAGAGTCGCCGTCGCCGATTTGCA	
RcCOL4-mu3-F	ACCCTCTCCGTCACGTGCGACCGAGAAATCCACTCT	For mutation of Cys-61
RcCOL4-mu3-R	CACGTGACGGAGAGGGTGGCGTCGTCGGCCTTGCA	
RcCOL4-mu4-F	ACGTCACGTCCAAGGCCGACGACGCCACCCTCT	For mutation of Cys-69
RcCOL4-mu4-R	GGCCTTGGACGTGACGTGGGCGGGAGCCTGCT	
RcCO-VIGS-F	TGAATCCGGTGAAGAACAGCAAC	VIGS vector construction
RcCO-VIGS-R	ACCTTCTCCATACTGAACTGGTAC	
RcCOL4-VIGS-F	GTTTCACTCGGGGCAATC	VIGS vector construction
RcCOL4-VIGS-R	TTG AGT CGT CCG AGT TGA GTG AG	
CORE-BOX(BJ)-F	GTTTATCTTTGAACAAAGATGAACCACAGAATGATCGATCAGCAGCAT	CORE motif probe
CORE-BOX(BJ)-R	ATGCTGCTGATCGATCATTCTGTGGTTCATCTTTGTTCAAAGATAAAC	
CORE-BOX-F	GTTTATCTTTGAACAAAGATGAACCACAGAATGATCGATCAGCAGCAT	Competitor probe
CORE-BOX-F	ATGCTGCTGATCGATCATTCTGTGGTTCATCTTTGTTCAAAGATAAAC	
RcGAPDH-F	GCTGGCAGGTATCCTTTCTG	q-PCR
RcGAPDH-R	GGCGACAATATCAGCCAAGT	
RcCO-Q-F	GATACCACCGAGGACGGGTT	q-PCR
RcCO-Q-R	CAGGACGCAGCCTCATCTTC	
RcCOL4-Q-F	GGCTTCGATCTCCGCTTCTG	q-PCR
RcCOL4-Q-R	TCAACTTCCTCGACGACCGA	
RcCOL5-Q-F1	CGCCTCCCGGAAAGCCTATG	q-PCR
RcCOL5-Q-R1	CCCGGCGCCGAGTTAAAGAT	
RcFT-Q-F	AGCTTGTGAGTTGTGGGTCT	q-PCR
RcFT-Q-R	ATTGGGAACCGCCCAAGAAA	
Actin2-F	GGTAACATTGTGCTCAGTGGTGG	RT-PCR
Actin2-R	AACGACCTTAATCTTCATGCTGC	
RcCO-RT-F	CTCCCCATCTCCGGCTTCCTC	RT-PCR
RcCO-RT-R	TCCATCCCCAACTGATG	
RcCOL4-RT-F	GTTCTGCCGAGCAGACTCCGC	RT-PCR
RcCOL4-RT-R	AGATCCAAATACGGATCCA	
